# *Olsenella uli*-induced pneumonia: a case report

**DOI:** 10.1186/s12941-022-00499-2

**Published:** 2022-03-02

**Authors:** Yufen Yan, Hong Li, Shuai Li, Shuhui Liu, Nan Jia, Yanfei Liu, Qing Liu, Jing Li, Chunhua Han

**Affiliations:** 1 Department of Outpatient and Emergency, the Affiliated Hospital of Qingdao University, 16 Jiangsu Road, 266000 Qingdao, China; 2 Department of Pharmacology, Qingdao University School of Pharmacy, 26 Ningde Road, 266073 Qingdao, China; 3 Department of Pathology, the Affiliated Hospital of Qingdao University, 16 Jiangsu Road, 266000 Qingdao, China; 4 Department of Clinical Laboratory, the Affiliated Hospital of Qingdao University, 16 Jiangsu Road, 266000 Qingdao, China

**Keywords:** *Olsenella uli*, Pneumonia, Human infection

## Abstract

**Background:**

*Olsenella uli* is anaerobic or microaerophilic bacteria, commonly found in oral cavity or gastrointestinal tract, which has not been reported to be associated with lower respiratory tract infection. Herein, we report the first case of *Olsenella uli* infection in the lung.

**Case presentation:**

A 70-year-old male farmer with no history of other respiratory tract diseases developed a cough with bloody sputum three times a day without obvious causes or other concomitant symptoms. After a period of treatment with empirical antibiotic, his condition did not improve. The computed tomography (CT) and lung biopsy results indicated bilateral pneumonia, and *Olsenella uli* was identified by micromorphology, sequence analysis and mass spectrometry analysis recovered from sputum. Ceftazidime, a third generation cephalosporin was used for the treatment, and the patient recovered after 10 days.

**Conclusions:**

Our report suggests a causative role of gingival bacteria in the pathogenesis of pneumonia, thus early diagnosis and prompt antibiotic therapy may play a role in the treatment of *Olsenella uli* induced pneumonia.

**Supplementary Information:**

The online version contains supplementary material available at 10.1186/s12941-022-00499-2.

## Background

Pneumonia is the most common infectious cause of mortality and morbidity worldwide. Pneumonia results from a complex process where the lower respiratory tract is infected with a causative microbial agents. It is estimated that lower respiratory tract infections cause 2.38 million deaths and an estimated 91.8 million disability-adjusted life-years [1]. Pneumonia can be acquired in the community or the hospital environment, and can be transmitted by the aspiration or inhalation of a pathogenic microorganism. Bacteria, viruses and fungi are the common pathogenic microorganisms causing pneumonia. With increasing antimicrobial resistance, pneumonia induced by known bacteria is a threat to human health [2]. In recent years, new bacteria or well-known bacteria such as *Rhodococcus defluvii*, *brucella* spp, which rarely infected the lung have been reported to cause pneumonia [3, 4].

The genus *Olsenella* is a group of bacteria that are aerotolerant anaerobe [5]. At the time of writing this manuscript, the genus includes six species, *Olsenella uli*, *Olsenella profusa*, *Olsenella umbonata*, *Olsenella scatoligenes*, *Olsenella timonensis* and *Olsenella faecalis* (www.bacterio.net/olsenella.html). *Olsenella uli* was originally designated as *Lactobacillus uli* in 1991 [6] and subsequently moved to a new genus, *Olsenella* based on genomic studies in 2001 [7]. *Olsenella uli* is regularly isolated from lesion sites in the human mouth such as gingival and subgingival sites with periodontitis [5, 7]. In 2008, Shane R Durkin et al. isolated *Olsenella uli* from vitrectomy specimen of an Australian man with chronic postoperative endophthalmitis [8]. Additionally, *Olsenella uli* was isolated from blood of humans with local oral or gastrointestinal infections [9, 10]. However, there are no reports on *Olsenella* infection in the lungs, with either culture or gene analysis. Based on clinical and laboratory work, we report the first case of *Olsenella uli* infection in the lung causing pneumonia, which may provide new ideas for interventions of *Olsenella uli* infection.

## Case presentation

**Fig. 1:**
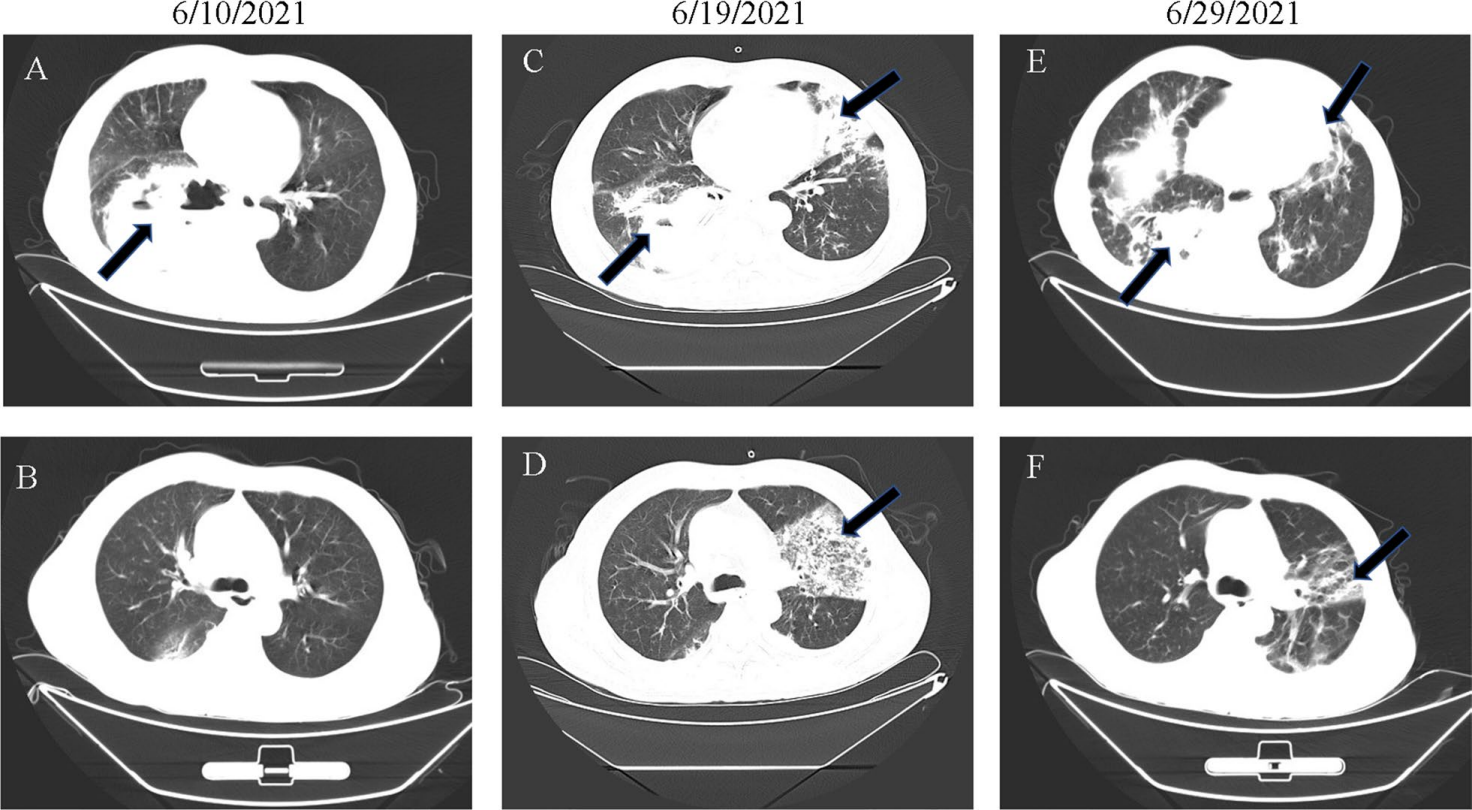
Computed tomography (CT) results of the *Olsenella uli*-induced pneumonia. Considering the day when the patient was transferred to our hospital as the first day, **A**, **B** CT on the second day(6/10/2021) showed bilateral pneumonia, pyogenic necrosis in the right lower lobe of the lung, encapsulated pyothorax in the right chest and bilateral emphysema, as the arrow indicated; **C**, **D** CT on the tenth day(6/19/2021), the arrows showed worsening bilateral pneumonia and emerging bilateral pleural effusion compared with the second day; **E**, **F** CT on the twentieth day (6/29/2021), arrows showed improvement in the bilateral pneumonia and left pleural effusion in comparison with the tenth day

A 70-year-old male farmer had a previous history of hypertension with peak blood pressure of 160/110 mmHg, but no history of diabetes, coronary heart disease, chronic lung disease, kidney disease and liver disease. He had a 30-year history of smoking 15–20 cigarettes per day and quit smoking 10 years ago. On May 31, 2020, he developed a cough with bloody sputum three times a day without obvious causes, which was not accompanied by fever, chest pain and distress, difficulty in breathing abdominal pain and diarrhea. After anti-infection and hemostatic treatment at the local hospital, the bloody sputum disappeared, but he developed difficulty in breathing, which was alleviated when lying on the right side, accompanied by fever with a peak of 38.9 ℃ on June 8, 2020. Since his condition did not improve, he was transferred to our hospital on June 10, 2020. The computed tomography (CT) results showed a mass shadow with uneven density, including gas and liquid density shadow in the lower lobe of the right lung, the density shadow of encapsulated liquid and gas, gas-liquid level in the right chest, multiple patchy shadows in both lungs, multiple vesicular translucent shadows in both upper lungs (Fig. 1A). These findings indicated bilateral pneumonia, pyogenic necrosis in the right lower lobe of the lung, encapsulated pyothorax in the right chest and bilateral emphysema. Routine blood test showed white blood cells 41.74 × 10^9^/L, neutrophil count 36.99 × 10^9^/L, neutrophil ratio 88.60%, lymphocyte count 2.14 × 10^9^/L, lymphocyte ratio 5.10%, platelets 533 × 10^9^/L. Abnormal liver function test results included Alanine aminotransferase (ALT) 61.3 U/L, Aspartate aminotransferase (AST) 46.5 U/L, Cholinesterase (CHE) 1229 U/L, Total bilirubin (TB) 36.3 umol/L, Direct Bilirubin (DB) 20.2 umol/L, Lactate dehydrogenase (LDH) 404 U/L. Abnormal renal function test results included Blood urine nitrogen (BUN) 8.71 mmol/L, Creatinine (Cr) 89.0 umol/L; Hypersensitivity C reactive protein (Hs-CRP) 175.35 mg/L. Abnormal coagulation function test results included Prothrombin time (PT) 16.40, international normalized ratio (PT-INR) 1.40, D-dimer (DD) 1040 ng/mL. Sputum anti-acid staining and HIV antibody (HIV-Ab) were negative (−). The patient was diagnosed as pneumonia with pulmonary abscess and underwent CT-guided drainage. Lung biopsy showed chronic inflammatory cell infiltration and focal alveolar epithelial hyperplasia in the right lung, which confirmed the CT results. According to the traditional experiences, biapenem combined with piperacillin and tazobactam was administered. On the eighth day, repeat chest CT plain showed worsening of the pulmonary lesion compared with the previous symptoms, suggesting that the treatment was ineffective (Fig. 1C, D).

**Fig. 2:**
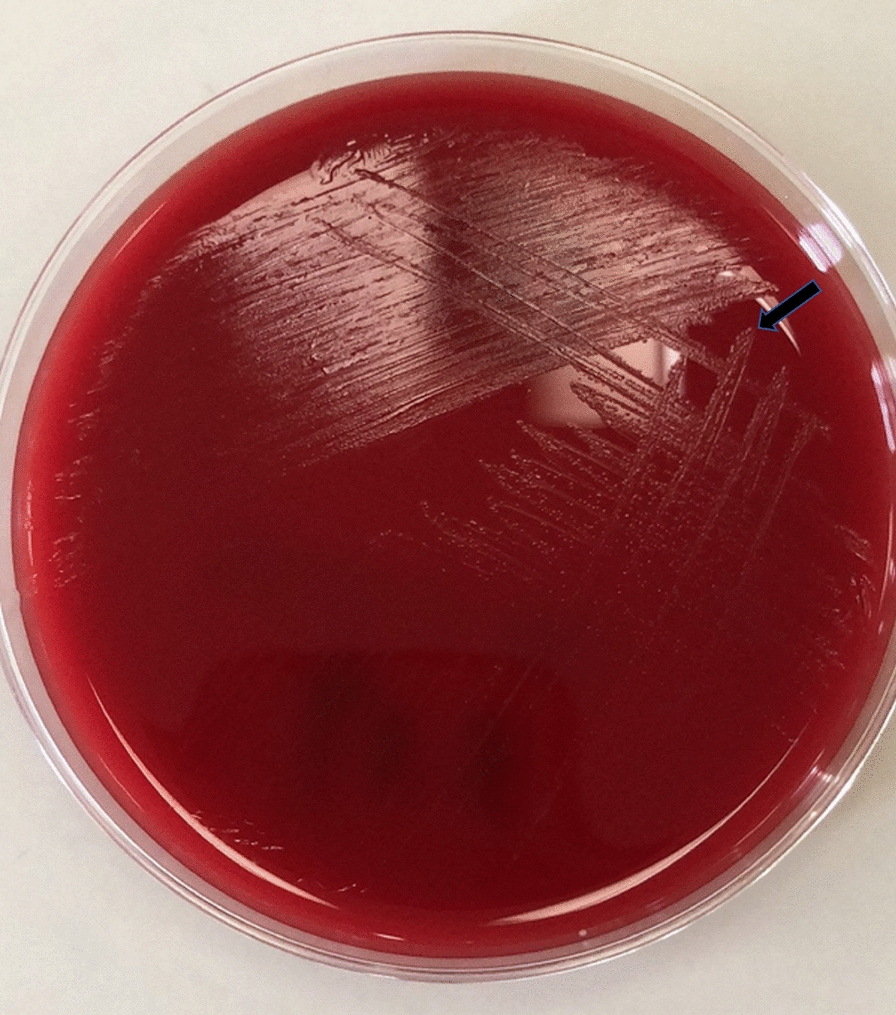
Colony morphology of *Olsenella uli* on Columbia blood agar medium. Colonies of bacteria isolated from sputum culture on Columbia blood agar were small needle tip like and translucent after 48 h of incubation at 37 °C with 5% CO_2_

**Fig. 3:**
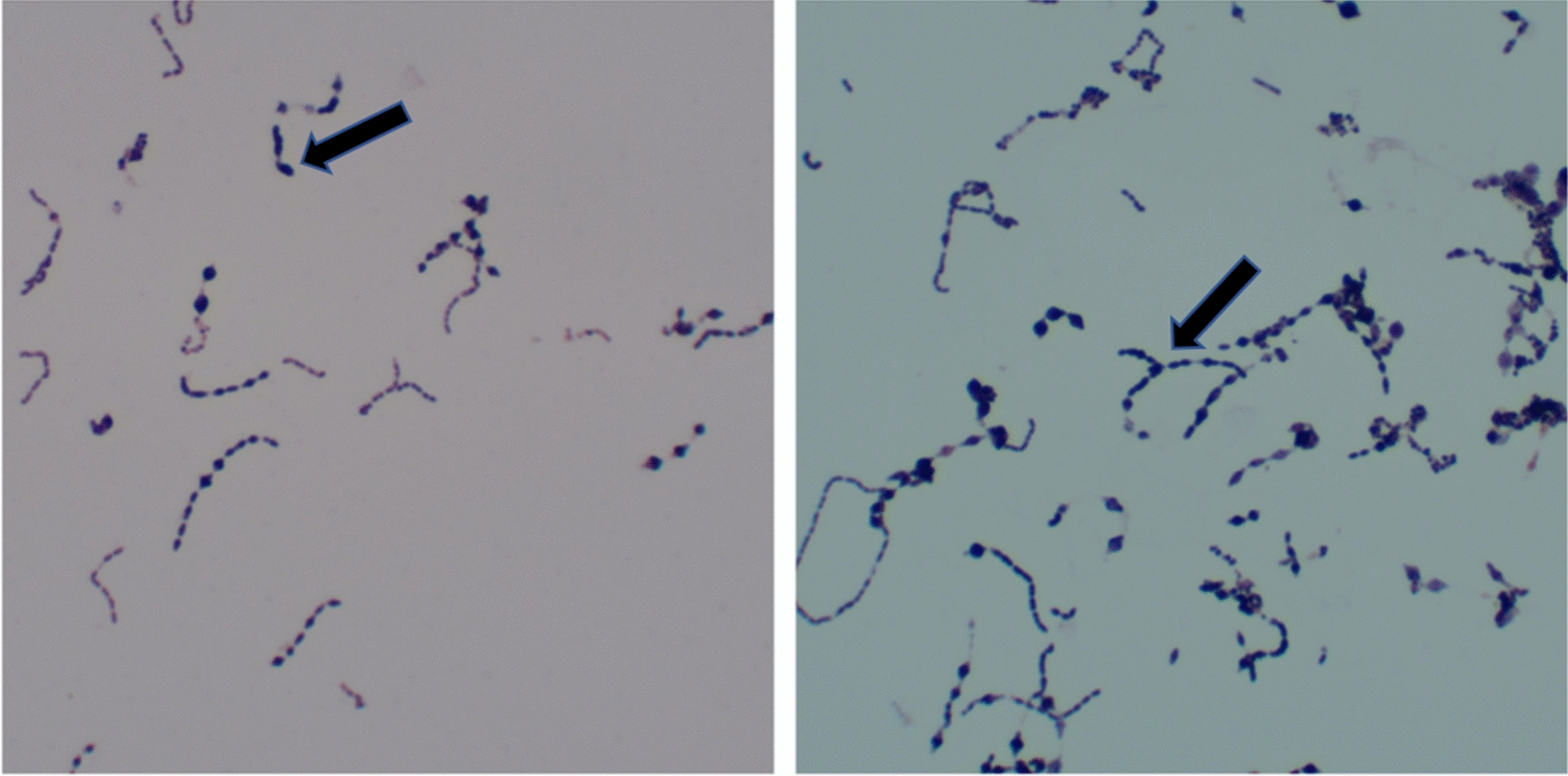
Microscopic morphology of *Olsenella uli*. After Gram-staining, gram-positive, purple cells appeared singly, in pairs, in short to long chains under microscope. The center of some cells was swollen, but the size was not uniform. Magnification ×1000

**Fig. 4:**
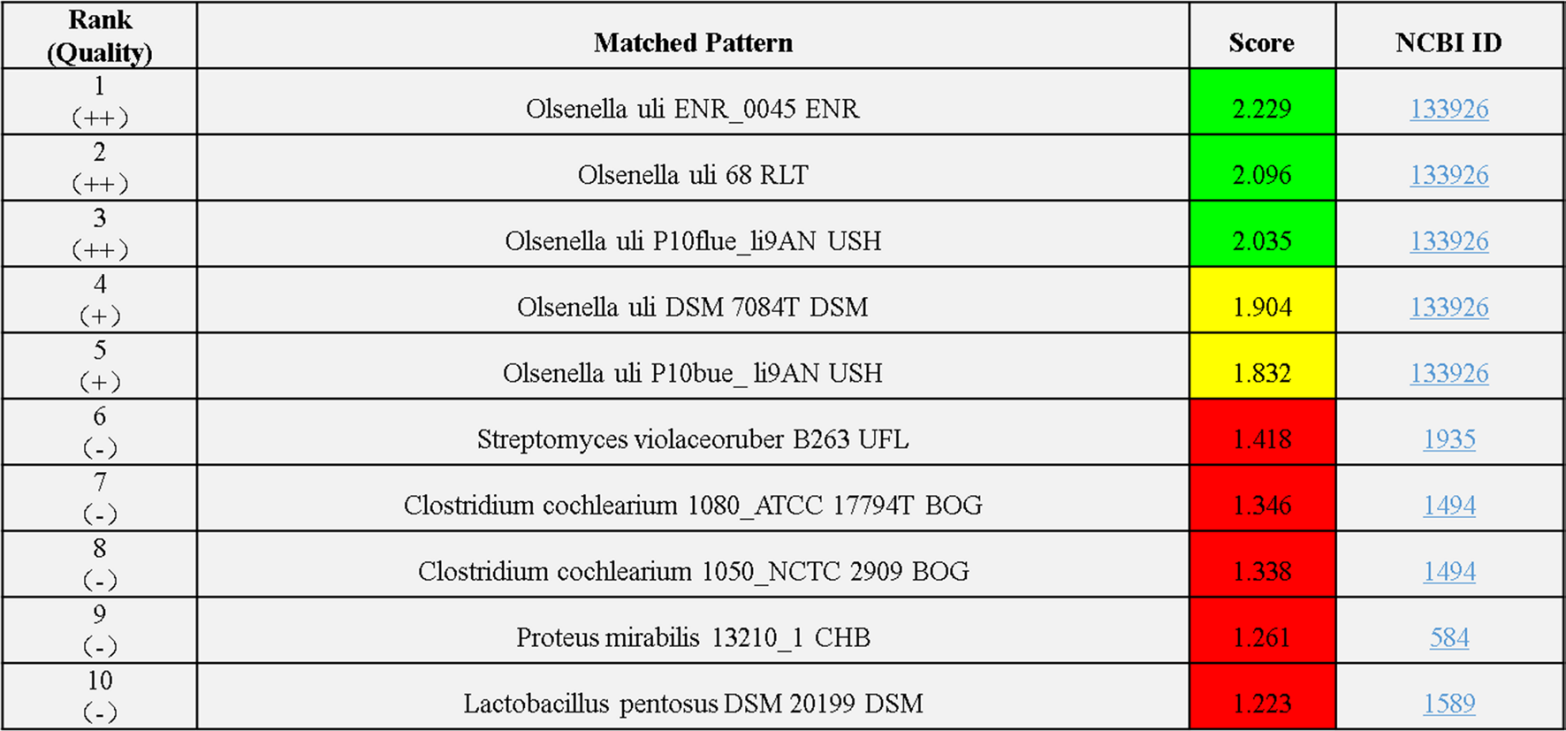
Results of mass spectrometry analysis for sputum culture. With Brooke mass spectrometer, the sputum culture of the patient was defined as *Olsenella uli*, with average score 2.019 ± 0.157

A Gram-positive bacillus was isolated from the sputum culture on the ninth day. Sputum samples were separately inoculated on Columbia blood agar, MacConkey agar medium and chocolate agar medium containing vancomycin. Colonies on Columbia blood agar were small needle tip like and translucent (Fig. 2) after 48 h of incubation at 37 °C with air plus 5% CO_2_. The microorganism was unable to grow on McConkey agar medium and chocolate agar medium containing vancomycin. Cells were analyzed under microscope after gram staining. Gram-positive, blue purple cells were seen singly, in pairs, in short or long chains, but the size was not uniform (Fig. 3). With Brooke mass spectrometer MALDI-TOF, the bacteria were identified as *Olsenella uli*, with average score 2.019 ± 0.157 (Fig. 4).

For further identification, two single colonies from the Columbia blood agar were selected. After genomic DNA extraction, the general primers [11] (27 F and 1492R, Table 1) for 16S rDNA were used for PCR amplification. Following gel electrophoresis, purification, recovery, cloning and sequencing, the sequencing results (GenBank accession numbers MZ220349 and MZ220350) were analyzed by BLAST, and the homology to the *Olsenella uli* sequence in the GenBank was 99%, and the NCBI number referenced was CP002106.1.

**Table 1:** Sequences of primers for 16 S rDNA Sequencing in this study

Primers	Sequence (5′–3′)	Product	Product size (bp)	Reference
27 F	AGTTTGATCMTGGCTCAG	16 S DNA	1500	[11]
1492R	GGTTACCTTGTTACGACTT			

As *Olsenella uli *is anaerobic or microaerophile, and sensitive to third generation cephalosporins [8], ornidazole combined with ceftazidime was used for the patient’s treatment. After 10 days, chest CT showed significant improvement in the pulmonary lesion and pleural effusion (Fig. 1E, F). As there were no clinical symptoms, the patient was discharged on the following day. With regular follow-up visits, the patient recovered fully from the pulmonary infection.

## Discussion and conclusions

In this study, we isolated *Olsenella uli* confirmed by mass analysis and sequence analysis from the sputum of a patient with pneumonia accompanied by pulmonary abscess. Colonies of *Olsenella uli* on Columbia blood agar were small needle tip like and translucent, with some differences from *Olsenella uli* type strain DSM 7084^T^ [5], which is semi-translucent or opaque after seven days of culture on FAA and PYG. The colonies of *Olsenella uli* from sputum shared more similarity with the *Olsenella uli* strain VPI D76D-27C^T^ [12]. Culture process and gram staining confirmed *Olsenella uli* as gram-positive, blue purple cells. Similar to *Olsenella uli* DSM 7084^T^ [5] and VPI D76D-27C^T^ [12], *Olsenella uli* isolated from the patient’s sputum appeared singly, in pairs and in short to very long serpentine chains, and the center of the cell was occasionally swollen, but the size was not uniform. Differences in colonies and appearance of *Olsenella uli* may result from the different disease sites or culture conditions. We used Columbia agar and aerobic condition (air plus 5% CO_2_, 48 h), which may have led to the atypical appearance of *Olsenella uli*.

Although there has been a significant decline, anaerobic bacteria remain the most common microorganisms in the lower respiratory tract [13], which results in aspiration pneumonia, followed by purulent pneumonia and finally lung abscess or complicated empyema. Anaerobic pneumonia infection is clinically characterized by fever, cough, stink and purulent sputum. The chest CT shows changes of pneumonia and lung abscess. *Olsenella uli*, which is anaerobic or microaerophile, has not been reported to be associated with lower respiratory tract infection. In this study, we reported the first pneumonia case induced by *Olsenella uli* with similar clinical and laboratory characteristics to anaerobic lung infections. This may provide new etiology in pneumonia, and requires further study.

*Olsenella uli* can be detected in the oral cavity or gastrointestinal tract by culture or molecular analysis [5, 7]. Interestingly, several studies have found that microorganisms colonizing the oropharynx, nasopharynx or upper gastrointestinal tract can be aspirated into the lung and cause aspiration pneumonia [14]. So, we hypothesized that our patient developed pneumonia after lung infection by aspirating *Olsenella uli* from the oral cavity or upper gastrointestinal tract. There are multiple risk factors for aspiration pneumonia, such as age, dysphagia, reduced consciousness, neurological disorders, gastroesophageal reflux disease, tube feeding, male gender, smoking, diabetes mellitus [14]. Our patient had three risk factors, age, male gender and smoking history, which supported our hypothesis along with the clinical characteristics such as fever, cough, sputum and imaging diagnosis of pneumonia and pulmonary abscess. Since there was no evidence of *Olsenella uli* in the oral cavity or gastrointestinal tract of our patient, the source and mechanism of *Olsenella uli* inducing pneumonia remained unclear. However, this report provides a new etiology for pneumonia especially with complicated pulmonary abscess, and may propose some more ideas for intervention of *Olsenella uli.*

Our report suggests a causative role of gingival bacteria in the pathogenesis of pneumonia, so early diagnosis and prompt antibiotic therapy may play a role in the treatment for *Olsenella uli* complex pneumonia.

## Supplementary Information

**Additional file 1.** Supplementary Document 1. Medical Ethics Committee of the Affiliated Hospital of Qingdao University (QYFY WZLL 26341).

## Data Availability

All data generated or analyzed during this study are included in this published article.

## Abbreviations

CT: The Computed Tomography
ALT: Alanine Aminotransferase
AST: Aspartate Aminotransferase
CHE: Cholinesteras
TB: Total Bilirubin
DB: Direct Bilirubin
LDH: Lactate Dehydrogenase
BUN: Blood Urine Nitrogen
Cr: Creatinine
Hs-CRP: Hypersensitivity C Reactive Protein
PT: Prothrombin Time
PT-INR: International Normalized Ratio
DD: D-dimer
HIV-Ab: HIV Antibody
