# Supplementary material for: Olsenella uli-induced pneumonia: a case report
Source: Ann Clin Microbiol Antimicrob. 2022 Mar 2;21:9. doi: 10.1186/s12941-022-00499-2 (PMC8889775; doi:10.1186/s12941-022-00499-2)
Supplement: Supplementary file 1 — Additional file 1. Supplementary Document 1. Medical Ethics Committee of the Affiliated Hospital of Qingdao University (QYFY WZLL 26341). [file 12941_2022_499_MOESM1_ESM.pdf]

The Affiliated Hospital of Qingdao University
Approval Document of Medical Ethics Committee

Approval number: QYFY WZLL 26341

Project name: The Olsenella uli induced pneumonia: A case report

Institution: The Affiliated Hospital of Qingdao University

Team leader: Chunhua Han

Project introduction:

This study intends to conduct microbial culture and analysis on sputum and other specimens derived from a patient with pneumonia who comes for medical care on June 10, 2020. In addition, clinical data of the patient would be analyzed, so as to accumulate experience for clinical diagnosis and treatment of such patients and promote the progress of medicine. In the whole study, we ensure the information of the patient including the name, gender, age, clinical data not leaked, and other rights and interests of the patients such as medical treatment are fully protected.

Opinion of medical ethics committee:

In this project, the rights and interests of the subjects are fully protected, which meets the requirements of the medical ethics committee. The protocol was agreed.

Medical Ethics Committee (seal)

June 11, 2020
